# Spatially Localized Visual Perception Estimation by Means of Prosthetic Vision Simulation

**DOI:** 10.3390/jimaging10110294

**Published:** 2024-11-18

**Authors:** Diego Luján Villarreal, Wolfgang Krautschneider

**Affiliations:** 1 Departamento de Mecatrónica y Biomédica, Escuela de Ingeniería y Ciencias, Instituto Tecnológico y de Estudios Superiores de Monterrey, Monterrey 64700, Mexico; 2 Institut für Integrierte Schaltungen, Hamburg University of Technology, D-21073 Hamburg, Germany; krautschneider@tuhh.de

**Keywords:** prosthetic vision, retina implant, single-cell selectivity, visual phosphenes, pixelized visual perception

## Abstract

Retinal prosthetic devices aim to repair some vision in visually impaired patients by electrically stimulating neural cells in the visual system. Although there have been several notable advancements in the creation of electrically stimulated small dot-like perceptions, a deeper comprehension of the physical properties of phosphenes is still necessary. This study analyzes the influence of two independent electrode array topologies to achieve single-localized stimulation while the retina is electrically stimulated: a two-dimensional (2D) hexagon-shaped array reported in clinical studies and a patented three-dimensional (3D) linear electrode carrier. For both, cell stimulation is verified in COMSOL Multiphysics by developing a lifelike 3D computational model that includes the relevant retinal interface elements and dynamics of the voltage-gated ionic channels. The evoked percepts previously described in clinical studies using the 2D array are strongly associated with our simulation-based findings, allowing for the development of analytical models of the evoked percepts. Moreover, our findings identify differences between visual sensations induced by the arrays. The 2D array showed drawbacks during stimulation; similarly, the state-of-the-art 2D visual prostheses provide only dot-like visual sensations in close proximity to the electrode. The 3D design could offer a technique for improving cell selectivity because it requires low-intensity threshold activation which results in volumes of stimulation similar to the volume surrounded by a solitary RGC. Our research establishes a proof-of-concept technique for determining the utility of the 3D electrode array for selectively activating individual RGCs at the highest density via small-sized electrodes while maintaining electrochemical safety.

## 1. Introduction

Phosphenes serve as the foundation for artificial vision [1]. Any visual sensation that is characterized as a spot of light in the visual field that is not brought on by light stimulating the visual system is called a phosphene. Several triggered phosphenes were reported to generate identifiable symbols and basic forms by Humayun et al. [2], Keserü et al. [3], Zrenner et al. [4], and Klauke et al. [5]. Doughnut-shaped structures [2], “round” patches of light [2,5], elongated shapes [3,5], lines [5], oval shapes [5], and more intricate patterns [5] have all been identified as phosphenes. Atypical patterns of retinal activity remain a concern in advanced retinal implants [6]. Electrical stimulation-induced perceptions are varied [2,3,5,7] and support only limited visual function [5,6]. As evidenced by ongoing clinical research, patients do not perceive a whole visual image consisting of phosphenes displayed simultaneously. In recent clinical studies related to Argus II developed by Second Sight Medical Products in the United States [6], 7 out of 30 test subjects demonstrated a maximum measured visual acuity of 20/1260. Comparable conclusions were recently drawn from the Alpha IMS created by Retina Implant AG in Germany, which revealed an achieved visual acuity of two test volunteers of 20/546 and 20/2000, respectively, out of nine test volunteers [8]. 

The topology of the electrode array is crucial since it determines the width and form of the phosphenes during electrical stimulation [9]. The electrical current spread varies in response to the electrode array architecture and active-ground electrode locations when specific stimuli are delivered. This may lead to the generation of phosphenes with forms other than a small point of light, causing modifications in the stimulation region. As artificial vision is still in the early phase of development, further research is necessary to fully comprehend the physiological underpinnings of prosthetic vision. In combination with achieving single-cell selectivity, both are mandatory for aligning retinal activation with a high stage of maturity. 

Attempts in the advancement of visual implants are based on the same concept of providing targeted stimuli by generating dot-like points of light or phosphenes [10]. Prosthetic devices with large electrode diameters of 500, 200, and 100 µm have been developed [5,6,8,11,12] with the goal of protecting fragile retinal tissue from irreversible reactions at the electrode interface [13]. The delivery of current from large electrodes is expected to activate a cluster of cells in the visual pathway, producing activity that differs from that of a stable retina and limiting detail perception [14]. Small-sized electrodes, however, are needed to accurately elicit small groups of RGCs and replicate typical retinal patterns [14,15]. For near-normal vision, the electrode density and diameter must be similar to the cell density and size [16]. Nevertheless, high charge density arises from the threshold current from small electrodes, damaging delicate retinal tissue [15]. As a result, a trade-off is identified between tissue protection and visual resolution. 

For a targeted stimulus, the distance of the electrode carrier to the RGC is key [9]. Close proximity lowers the threshold current and charge density, resulting in well-defined stimulation. In contrast, distant proximity contributes to the current distribution, increasing the stimulation of packages of RGCs, an increase in volume stimulation [9], and a compromised visual resolution. Fine et al. [17] stated that as the current amplitude of a pulse increases, gradually more cells under the electrode will reach the depolarization threshold. The ideal scenario for visual prostheses would be to faithfully replicate the spatiotemporal activity patterns that naturally occur in retinal ganglion cells (RGCs). This implies that every electrode must selectively activate single cells. RGCs are mostly located near the fovea and are densely packed in the ganglionic layer. Temporal patterns of activity in spatially mixed RGC types convey unique features of the visual world to the brain. As a result, nearby RGCs typically emit different signals from one another [18]. Existing approaches to single-cell selectivity have been shown to be insufficient [6,8]. Each electrode should stimulate an adjacent cell to produce a small amount of activated light that is useful as a building block for the pattern of phosphenes [9]. 

Retinal prostheses typically send stimuli through a planar surface electrode array with the expectation of triggering single RGCs selectively [14,18]. In recent studies of photomicrographs of healthy retinas from monkeys [19], mice [20] and humans [10,21], the highest volumetric density of RGCs was shown to prefer the center of the ganglionic layer along the vertical section, leaving only a few RGCs near the vitreous medium boundary. This implies that eliciting RGCs in relatively close proximity can lead to a misinterpretation of the natural spatiotemporal patterns of cells. A realistic restoration of typical RGC activity is projected to include the specific and independent activation of solitary RGC forms. In contrast to clinical prostheses, recent experimental studies suggest that a higher electrode array density and a smaller electrode diameter can individually trigger RGCs in isolated retinas [14,22]. Retinal prostheses face significant challenges, i.e., the maldistribution of current delivery through sizeable electrodes, resulting in multiple stimulated phosphenes with shapes other than a narrow spot of light and targeting single RGCs at high densities [8,23]. Even so, detailed and isolated cell triggering requires high electrode array density, novel electrode array design, and more sophisticated stimulation patterns [24,25].

This work examines the effects of two independent electrode array topologies to achieve a single-localized stimulation: the 2D hexagon-shaped surface array reported by Klauke et al. (2011) and a patented 3D linear electrode carrier. For the hexagon-shaped array, an algorithm was developed that estimates the experimental shape and breadth of phosphenes found by Klauke et al. [5] on the basis of specific patterns of electrode stimulation. For the 3D linear electrode carrier [26], we used fine-scale spatial patterns of activation to provide a theoretical proof-of-concept for selectively activating individual RGCs at the highest density via small-sized electrodes while maintaining electrochemical safety. The latter is based on the following premises: (1) the use of the criterion of the volume of activation between two adjacent face-to-face electrodes and the assumption that states that if the current density from the electrodes reaches the RGC threshold, it can be assumed that a single or small group of RGCs will be triggered; (2) activating single cells using single electrodes could produce single-cell resolution and increase visual resolution [18]; (3) electrodes activating only nearby RGCs ensure that the perception produced by stimulation will be small (on the order of the electrode) and associated in visual space with the position of the electrode site on the retina (or pixel-wise stimulation) [25]; (4) to accurately replicate the natural spatiotemporal patterns of activity in RGCs of various kinds, an ideal prosthesis would need to be able to individually activate each RGC [18]; (5) unique features of the visual space are conveyed to the brain by temporal patterns of activity of spatially mixed RGC types [18], where thresholds for electrical stimulation are similar in all five typical RGC types (ON and OFF midget, ON and OFF parasol, and small bistratified ganglion cells) [14]. Across these topologies, we ask which would provide a well-defined localization of the stimulus using small electrodes and a safe stimulus current to target very near and deep RGCs in the ganglion layer? A detailed explanation is given in the Discussion. 

## 2. Materials and Methods

### 2.1. Two-Dimensional Hexagon-Shaped Surface Array

**Figure 1:**
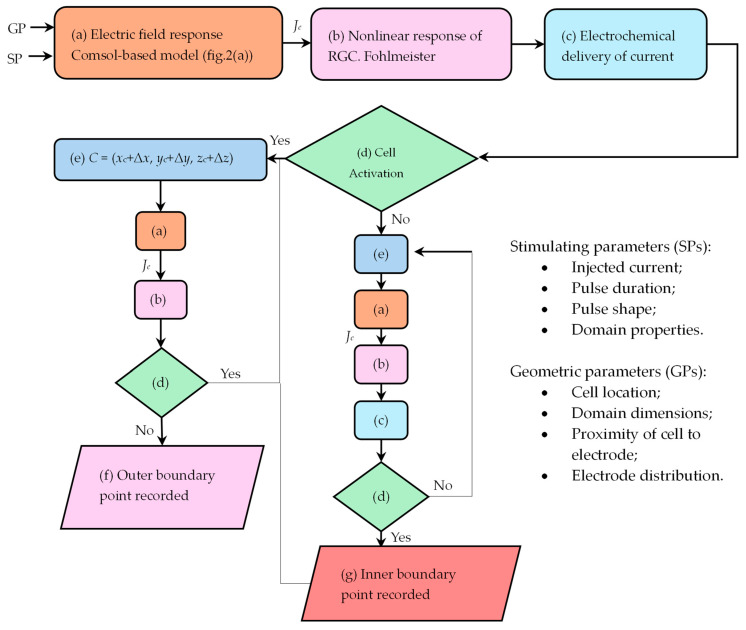
Algorithm of the activation area. Steps showing letters and colors that are related to the same corresponding actions in the algorithm. *J_c_* is the peak boundary current density of RGC. (Reprinted with permission from ref. [27] Copyright 2019, Springer Nature).

The algorithm for the hexagon-shaped array is displayed in Figure 1 (extracted and modified from [27]). The result is described as the region of the retina where certain cells respond to a stimulus applied to the ground electrode. The size and form of the phosphenes are determined, as well as the approximate number of active cells, by examining the stimulus criterion, which is defined as the space on the retina where the stimulus initiated at the active electrode triggers a response in some cells. Otherwise, the cell is not activated by this electrode.

#### 2.1.1. Response of the Electric Field 

**Figure 2:**
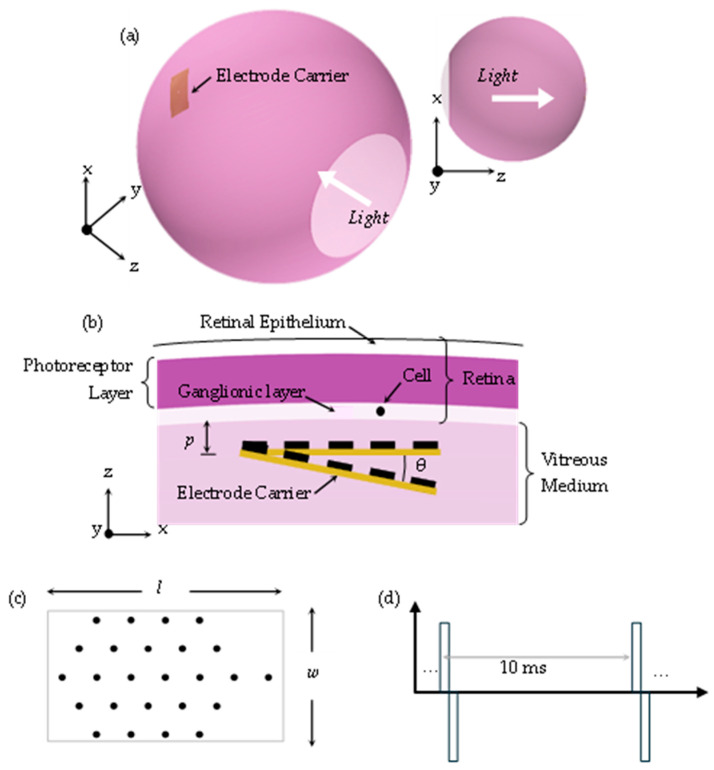
(**a**) Simulation model built in Comsol Multiphysics. (**b**) a zoomed-in view of the layers included in the model. p corresponds to the proximity of cells to the electrodes. θ is the angle of displacement along the width of the array. The electrodes are in contact with the retinal surface layer. (**c**) Twenty-five stimulating electrodes are arranged in a hexagonal array (data from [5]). (**d**) Charge-balanced biphasic current pulse with pulse train frequency of 100 Hz. (Reprinted with permission from ref. [27] Copyright 2019, Springer Nature).

A three-dimensional computational model for electrical stimulation was developed via COMSOL Multiphysics (Version 6.0, Comsol, AB., Stockholm, Sweden). The realistic 3D spherical reconstruction of the retina, which represents a portion of the human eye, is illustrated in Figure 2a. Table 1 and Table 2 include a list of geometric parameters and description values [28,29]. To induce single or nearby RGC stimulation, biphasic pulses of uniform current are supplied from the active electrode to the ground electrode. The voltage across the electrodes and the current provided by the active electrode were retrieved.

#### 2.1.2. Electrochemical Safety and Cell Neuron Nonlinear Response

RGC responses to electrical stimulation were investigated in COMSOL Multiphysics by adding the characteristics and equations that define the kinetics of the ionic channels into the RGC circuit model [30]. The peak boundary current density across the cell membrane was assumed to be the input parameter. Recent studies have employed this modeling assumption for axonal activation [31] and RGC stimulation [9]. The circuit model retains all of its information from the original model [30]. Safety associated with avoiding water electrolysis, corrosion development, and excessive tissue heating was considered during electrical current injection. 

A MATLAB version R2023a (MA, USA) script structured the retrieved data of the voltage across the electrodes and current given to the electrode, as well as performing many tasks to calculate the device’s heat dissipation and charge density on the electrode. The local charge density was calculated by integrating the current of the active electrode over time and dividing by the cross-section area of the electrode. Neural heating from the retinal implant was calculated as described in [32].

#### 2.1.3. Cell Shifting and Recording of Boundary Point of Activation

Relating to Figure 1 and Figure 2, the shifting of the cell and the recording of the boundary points of activation are explained as follows. Before proceeding, let us discuss some boundaries. For the procedure to function properly, the initial position of the cell (x_c_, y_c_, and z_c_) should be such that the cell is located inside the ganglion layer, immediately below the center of the active electrode. Put differently, the cell is placed concentric to the active electrode, seen from the *xy* plane (see Figure 2b). 

Stimulating parameters (SPs) and geometric parameters (GPs) are inputted in step (a) of the algorithm, see Figure 1, to obtain the electric field and the peak boundary current density of RGC, *J_c_*, using the time-dependent Electric Currents Module in COMSOL Multiphysics. Step (b) uses *J_c_* as an input to drive the stimulation of the RGC Fohlmeister [30] circuit model. If activation is achieved, the electrochemical safety is tested (step (c) in Figure 1). 

Two scenarios occur after step (d). When activation occurs at the initial position, the cell is shifted inside the ganglion layer (Δx, Δy, Δz) (step e), and steps (a) and (b) are executed until there is no activation and the outer boundary is identified. If activation is not achieved at the initial position, two cases arise: a percept such as a ring-shaped geometry can be formed, or there is no stimulation. 

In the former case, the cell is shifted inside the ganglion layer, and steps (a), (b), and (c) are repeated until there is activation and the inner boundary is found. Then, we continue with the outer boundary process. In the latter case, an increase in the injected current must be implemented. The procedure is run until the percept is completed. 

The closed geometry obtained from this algorithm is known as the stimulus criterion, defined as the space on the retina when a stimulus at the active electrode causes a response in certain cells. The output obtained from this algorithm can be characterized as geometries of light in the visual field known as phosphenes or visual sensations. For more information related to this algorithm, supporting evidence, and limitations, see [9].

#### 2.1.4. Parameter Settings for Stimulation

The model included stimulation settings as shown in [5]. For all subjects, the pulse train frequency was held constant at 100 Hz. 

The electrodes were stimulated with a single charge-balanced biphasic current pulse: see Figure 2d. A hexagonal array of 25 stimulating electrodes with a 100 µm electrode diameter and 500 µm center-to-center distance was used. The array was rebuilt in a 2 × 4 mm^2^ substrate and inserted for epiretinal stimulation. 

The nearby electrodes were triggered separately in every simulation, with a pulse train frequency of 100 Hz. That is, a 10 ms interval is sufficient for the membrane voltage to revert to its resting potential. The total evoked phosphene is the sum of the phosphenes induced by each pair of electrodes. Experimental evidence indicates that brightness fading has two temporal components: rapid fading between <0.5 s (5–60 Hz stimulating rate) [33] and 0.18 s (16 Hz rate) [34] and a slower reduction in brightness between several seconds (5–60 Hz rate) [33] and 14 s (16 Hz rate) [34]. 

According to Freeman [35], the gradual decrease in the firing rate may contribute to the decrease in brightness. This shows that the stimulation rate of 100 Hz in [9] elicited percepts that remained during the entire stimulation time of 1.5 s.

### 2.2. Three-Dimensional Electrode Carrier and Stimulus Criterion

The 3D electrode carrier is designed as an array of linear carrier elements (LCEs) that carry a plurality of electrodes positioned in a substantially straight line penetrating the ganglionic surface layer [26], 

A three-dimensional computational model developed in Comsol Multiphysics (Version 4.4, Comsol, AB., Stockholm, Sweden) was applied for the electric field response and cell activation. Two linear electrode carriers were included at the horizontal meridian in the superior direction, 1 mm away from the foveal center. This value is equal to a RGC density and distribution of 31,300 mm^−2^ [36], a ganglionic layer thickness of 60 µm [37], and a RGC diameter of 10 µm [38,39]. Photomicrograph data of the vertical distribution [10,19,20,21] were fitted to a 3rd-order polynomial, as shown in [40], and built such that the integral of the polynomial produces the realistic amount of RGCs per mm^2^, measured by [36]. The mathematical method states that the volume of stimulation produced by the current density distribution must be equivalent to the volume enclosed by a single cell, v = ρ_v_^−1^, where ρ_v_ is the volumetric cell density in µm^−3^. The volumetric cell density at the vertical distribution is computed as explained in [40]. The stimulus criterion states that if the activation of an electrode is accomplished within its volume, the RGC is stimulated. Otherwise, it is not. Let us agree that the RGC distribution per volume is homogenous and that volume can be characterized as a cube of equal length, λ = v^1/3^, where λ represents the proximity of the adjacent face-to-face electrode. As a result, in order to achieve single-localized stimulation, the current density distribution must satisfy the triggering of RGCs within the cube of simulation. A segment of the human eye was modeled containing tissue boxes, and was built to a greater degree of structural similarity than earlier published studies [28,29,41,42]. The electrical parameters and sizes of each layer are listed in Table 3 [28,29,41,42]. An assembly of a pair of active and ground electrodes was realized via an epiretinal design. A single, monophasic linear decrease pulse shape of 100 µs was injected to drive the RGC stimulation. The distribution of the extracellularly applied current density was dispersed along the tissues and the contributing channels observed in the RGC membrane. In this research, the membrane model defining the kinetics of voltage-gated ionic channels [30] was executed in COMSOL Multiphysics. The boundary current density in the RGC membrane computed by COMSOL was assumed to be the extracellular current in the RGC circuit modeling. Recent stimulation models have applied this modeling assumption for axonal activation [31] and RGC stimulation [9]. The threshold current for extracellular stimulation must produce a voltage shift of approximately +30 mV in the membrane. Retinal network cells (i.e., bipolar, horizontal, and amacrine cells, and ON and OFF networks) were omitted because the visual phototransduction process cannot be driven by severe rod and cone photoreceptor impairment. An electrode diameter of 7.5 µm was used. This dimension has been commonly used in ganglion cell stimulation [18,43,44,45]. A single RGC is placed between two linear carrier elements. This is set such that an equal length is present from the electrodes and the RGC. A single stimulation was examined by increasing the peak stimulation amplitude with an interval of 0.1 nA until the action potential was produced. The safety in terms of electrical performance is related to the charge density injection level of the mC/cm^2^ limit, as shown in [9].

## 3. Results

### 3.1. Three-Dimensional Electrode Carrier

**Figure 3:**
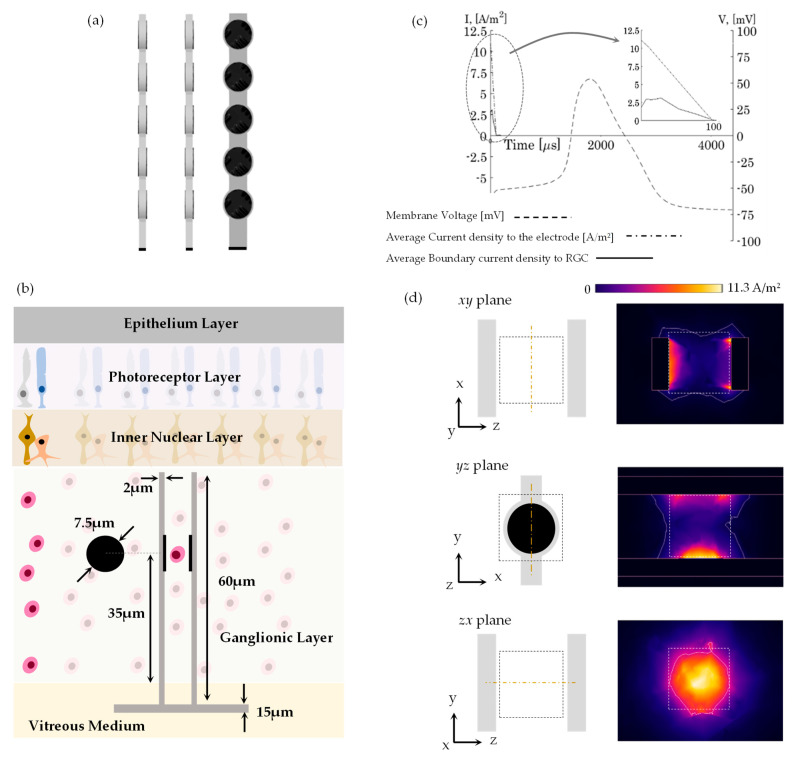
(**a**) A Three-dimensional linear electrode carrier can carry a plurality of penetrating electrodes. The electrodes are arranged along a substantially straight line to penetrate into or through the surface of the ganglionic layer. (**b**) The 3D retinal model implemented in Comsol Multiphysics (not drawn to scale). The model consists of tissue boxes that represent a segment of the human eye. A single pair of electrodes involving an active electrode and ground electrode is implemented using epi-retinal design. (**c**) The results of the action potential at the membrane triggered an average peak stimulus density of 11.31 A/m^2^ from the electrode. The single RGC located between the active and ground electrodes obtains an average boundary-peak stimulus density of 3.1 A/m^2^. (**d**) On the left, groups of planes (zx, xy, yz) illustrate dashed-line squares representing the stimulation cube (drawn to scale with respect to the size of the electrode). On right, the white-line squares represent the dimensions of the cube seen on the groups of the planes (drawn to scale with respect to the size of the electrode). Current density distribution was determined in COMSOL Multiphysics using the surface/contour feature. On the top, we used a colormap to identify current densities applied to the RGC.

Figure 3a shows two 3D linear electrode carriers can carry a plurality of penetrating electrodes. Electrodes are arranged along a substantially straight line to penetrate into or through the surface of the ganglionic layer. 

Figure 3b depicts a sketch of the layers of the retina along the vertical section. The linear carrier element length is used for positioning inside the ganglion layer. In this study, the penetrating electrodes were placed at the highest cell density, 1 mm away from the foveal center in the superior direction. This location is equal to 31,300 mm^−2^ RGC area density [36], 60 µm ganglion layer thickness [37], and 10 µm RGC diameter [38,39]. Villarreal [40] provided a 3rd-order polynomial that described the volumetric density as a function of the ganglion layer thickness and the RGC area density. Using those values, they indicated that the active and ground electrodes are located face-to-face with a peak volumetric cell density of 8.05 × 10^−4^ µm^−3^. The cube volume explained earlier is 1.24 × 10^3^ µm^3^, and the cube length (or inter-linear carrier length) is 10.75 µm. 

Figure 3c illustrates the action potential of a single RGC (dashed line) initiated with a single monophasic linear decrease pulse shape with a peak average current density of 11.3 A/m^2^ from the electrode (dashed-point line). This produces a threshold peak stimulus amplitude of 0.5 nA and a change density injection of 5.66 × 10^−5^ mC/cm^2^, assuming a pulse width of 100 µs. Figure 3c, top right, shows a zoomed-in view of the linear decrease pulse shape until a final time of 100 µs. 

For effective stimulation, a single RGC positioned concentrically between the electrodes (as seen from the xy plane) has a peak average boundary current density of 3.1 A/m^2^. 

Figure 3d, on the left, shows groups of planes (zx, xy, yz) and dashed-line squares representing the cube of stimulation (drawn to scale with respect to the size of the electrode). On the right, white-line squares represent the dimensions of the cube seen on the groups of planes (drawn to scale with respect to the size of the electrode). Current density distribution seen in Figure 3d, on the right, was determined in COMSOL Multiphysics using the surface/contour feature. On the top, we used a colormap to identify the current densities applied to the RGC.

### 3.2. Two-Dimensional Hexagon-Shaped Surface Array

**Figure 4:**
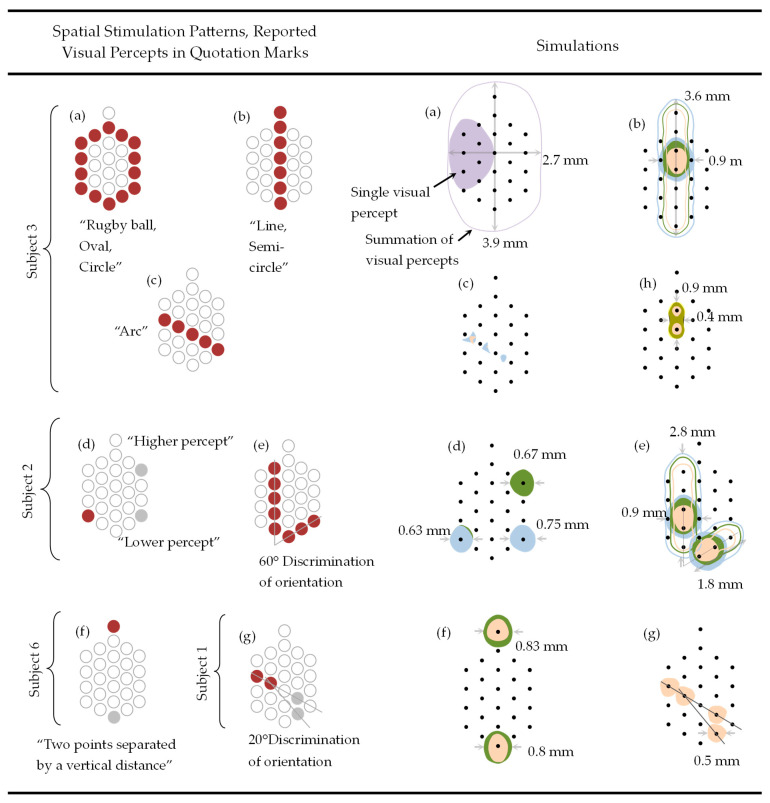
Relationship between stimulation patterns and simulations. Spatial patterns in the first column are drawn to scale with respect to electrode diameter and inter electrode distance. Simulated visual percepts highlighted in dark brown (in background), yellow, and orange in figure (**h**) are generated with the current amplitudes and proximities of 13 µA and 25 µm, 26 µA and 50 µm, and 13 µA and 50 µm. Conductivities of the ganglionic layer and intracellular space were decreased with a factor of 10 due to the different data found. Sizes of visual percepts are related to the surface of the retina. (Reprinted with permission from ref. [27] Copyright 2019, Springer Nature).

Figure 4 shows the spatial stimulation patterns and the reported visual percepts according to Klauke et al. [5] on the first column, and the results of our simulation using the 2D hexagon-shaped array on the second column. The first column is separated by subjects 1, 2, 3, and 6 (according to [5]) included in this study. The subjects’ reported visual percepts are shown below each spatial stimulus pattern. The peak current amplitude and pulse width were held constant at 13 μA and 94 μs, except for in Figure 4h, where our algorithm was tested for dot-like stimulation using 13 µA and 26 µA.

The spatial patterns from Figure 4a–g are linked to their respective results in the second column, except Figure 4h, which depicts the potential of restoring visual perception by adjusting the closeness, the conductivity surrounding the RGC, and the peak current amplitude. 

The spatial stimulation patterns for subject 3 shown in Figure 4a–c are organized in the first row. A set of two adjacent electrodes colored in red were used to obtain a set of adjacent active and ground electrodes that stimulated the retina and generated a single visual percept. This approach was repeated until all electrodes functioned as the active electrode. The summation of single visual percepts is linked to the second column.

The spatial stimulation patterns for subject 2 shown in Figure 4d,e are arranged in the second row. The former used an individual set of active (red) and ground (gray) electrodes for obtaining the “Higher percept” and the “Lower percept”. The latter used the same technique explained earlier relating to subject 3.

Subjects 6 and 1 are organized in the third row shown in Figure 4f and Figure 4g, respectively. Both used a set of active (red) and ground (gray) electrodes to stimulate the retina.

The evoked percepts colored in purple, blue, green, and orange (second column) are associated with 10, 100, 150, and 200 µm of closeness in Figure 4a, Figure 4b, and Figure 4d–g, respectively. The blue and green evoked percepts in Figure 4d correspond to lower and higher percepts, respectively. The blue and orange percepts in Figure 4c correspond to displacement angles with array widths of 2° and 4°, respectively. All the results met the electrochemical safety standards.

## 4. Discussion

### 4.1. Practical Context of Stimulation Differences Between 2D and 3D Arrays

RGC precise stimulation is discussed using the question from the Introduction: which would provide a well-defined localization of the stimulus using small electrodes and a safe stimulus current to target very near and deep RGCs in the ganglion layer? 

For this question, we used the results of the 2D hexagon-shaped array (Figure 4) on the basis of specific patterns of electrode stimulation, and the proof-of-concept of the 3D array (Figure 3) for selectively activating individual RGCs. Observing the results of the 2D array, our simulation was directly compared with the visual sensations induced by stimulating electrodes reported in clinical studies. The large variety of visual reactions previously described is strongly associated with our simulation-based findings, providing insight into the underlying brain processes and allowing for the development of analytical models of evoked percepts. Nevertheless, a localized dot-like visual sensation is only achieved using current amplitudes and proximities of 13 µA and 50 µm, respectively. Similarly, state-of-the-art 2D retina implants can only provide round-like experiences very near to the electrode, making the precise activation of deeper ganglion cells hard to achieve. Moreover, the displacement angle caused by natural eye movements could have an effect on RGC activation, particularly for single cells; see Section 4.3. 

Recent photomicrographs of healthy retinas from monkeys [19], mice [20], and humans [10,21] revealed that the maximum volumetric density of RGCs prefers the center of the ganglionic layer along the vertical section, leaving just a few RGCs near the vitreous medium border. This means that evoking RGCs in close proximity might cause a misinterpretation of the cells’ normal spatiotemporal patterns. A genuine restoration of normal RGC activity is expected to entail the selective and independent activation of solitary RGC forms. Unlike clinical prosthesis, current experimental research indicates that larger electrode array density and smaller electrode diameter can individually activate RGCs in isolated retinas [14,22]. Retinal prosthesis confronts considerable problems, such as the misallocation of current flow through large electrodes, resulting in many stimulated phosphenes with forms different to a condensed area of light [8,9,10,11,12,13,14,15,16,17,18,19,20,21,22,23]. Detailed and remote RGC activation necessitates high electrode array density, innovative electrode array design, and increasingly complex stimulation patterns [24,25].

The 3D array shows a promise for detailed localized activation. To accomplish that, the current density distribution must be sufficient to stimulate RGCs within the stimulation volume created by the current density distribution. It can be assumed that a single RGC and its volume can potentially fit between two electrodes of neighboring face-to-face linear carrier components. That is, the cube volume is around 1.24 × 10^3^ µm^3^ and the cell volume is around 0.523 × 10^3^ µm^3^. The ratio of cube volume to the cell volume is around 2.4, which signifies the possibility of a solitary RGC being placed within the electrodes. As a result, if the current density from the electrodes reaches the RGC threshold, it can be assumed that a single or small group of RGCs will be triggered. The visual perception produced by stimulating only nearby RGC is small (in the order of the electrode) and associated in visual space with the position of the electrode site on the retina (or pixel-wise stimulation) [25]. Typical retinal patterns could be replicated by accurately eliciting small groups of RGCs via small-sized electrodes [14,15]. The results indicate that a single RGC can be activated using a 7.5 µm electrode diameter and a very small stimulus amplitude of 0.5 nA, producing a change density of 5.66 × 10^−5^ mC/cm^2^, assuming the injection of a 100 µs monophasic linear decrease pulse shape. This indicates that a small number of RGCs could be activated at the highest density using a low and safe stimulus current and small-sized electrodes while maintaining electrochemical safety. Further, 3D-stimulation could enable the well-defined localization of stimuli anywhere in the retina, ensuring the precise activation of extremely close and deep ganglion cells and clear visual sensations, because small threshold currents are required. 

### 4.2. Proximity Between RGCs and Electrodes (2D Hexagonal Array)

The proximity of RGCs to electrodes affects the form and breadth of perceptions [9]. Low stimulus amplitudes can activate body cells in close proximity. This results in small areas of stimulation on the retina. Increasing proximity leads to a larger region of activation because the stimulus amplitude must rise to activate some RGCs, hence extending the size of the phosphenes [9]. However, in this investigation, the closeness of the RGCs to the electrodes increased, but the peak current amplitude remained fixed. This causes the current to gradually become inadequate to elicit a response from RGCs. As a result, the evoked visual percept shrinks as closeness increases (see Figure 4b,e,f).

### 4.3. Electrode Array and Displacement Angle (2D Hexagonal Array)

Epiretinal implants are placed close to the retina’s surface to achieve lower activation thresholds and a more precise stimulating region [9,28,29]. On the other hand, natural eye movements may reposition the electrode array in the vitreous cavity [46], dislocating the array at an angle that alters the direction of stimulation. The eyes often shift positions at a rate of two–three times per second [35]. This would have an effect on RGC activation, particularly for solitary cells. In this work, the electrode array was moved at angles of 2° and 4°, with the electrodes being 200 µm apart. This modest movement was enough to change the activation region provided by the electrode stimulation, resulting in complex percepts such as an arc, triangle, and egg form (Figure 4c). The current spread is unlikely to provide a symmetric stimulating area on the retina since parts in the electrode array are closer to the target neurons. This will cause alterations in the region of stimulation, resulting in percepts with forms different to round dot-like phosphenes. These findings indicate that a highly sensitive reaction to stimuli is present, affecting the aim of visual prostheses reaching a similar spot of light.

### 4.4. Stimulus Pattern Orientation (2D Hexagonal Array)

The appearances of the induced phosphenes are proportional to the spreading direction of the current density, which follows the location of the active electrode to the ground electrode [9]. This results in differences in the region of stimulation surrounding the stimulating and return electrodes (Figure 4d,e,g). The fact that visual percepts arise in the surroundings of the active and ground electrodes is not surprising because of the close proximity of the targeted RGCs. This could, however, be invalid for large angles of displacement and isolated proximities between RGCs and electrodes.

### 4.5. Degenerate Retina and Electrode Topology (2D Hexagonal Array)

Electrode array architecture and electrode characteristics affect the form and width of phosphenes. Simulation-based discoveries in [9] demonstrated that single and multiple topologies of stimulating electrodes produce diverse percept forms, such as marked and circular shapes. A probable explanation for such shapes is that the ground is far from the active electrode, generating deep current flow into the tissues and increasing the number of targeted RGCs. Experiments with various electrode topologies revealed diverse forms of phosphenes other than a small spot of light [2,3,5]. The cell density in the degenerating retina determines perceptual shape and development. Keseru [3] reported that thresholds differ with experimental stimulation, which was attributed to variable degrees of retinal degeneration. This can have an impact on the structure of visual perception because gaps in dark vicinities may appear in places of cell depletion [9].

### 4.6. Peak Stimulus Amplitude (2D Hexagonal Array)

The breadth of elicited percepts and the amount of peak current amplitude are well associated. That is, far-localized RGCs can be activated by increasing the injected current. Experimental findings by Rizzo [11] in human volunteers revealed a direct relationship between the stimulus charge and the size of the percept. Figure 4h demonstrates that the elicited percept can be restored by doubling the peak stimulus, despite having different proximities.

### 4.7. Phosphene-Based Prosthetic Vision

Visual prostheses produce phosphenes by stimulating the visual cortex, the optic nerve, or the retina along the visual pathway. Recent psychophysical research has shown that visual prostheses can improve visual perception and function. Perez-Yus [47] used computer vision algorithms to elicit phosphene-based stimuli with semantic significance from real-world indoor surroundings. Sanchez-Garcia [48] investigated image identification via a convolutional neural network to comprehend various interior scenes under prosthetic vision constraints. Denis [49] created a real-time text localization method to enhance text accessibility. Guo [50] applied contrast enhancement under simulated prosthetic vision, edge detection, and zooming techniques to greatly increase object identification accuracy. Abraham [51] recently employed active photonic sensing for picture scanning and text size adjustment to improve word recognition rates.

### 4.8. RGC Activation by Means of a 3D Electrode Carrier

The 3D electrode carrier presented in this analysis is positioned at horizontal meridians (nasal and temporal) in the superior orientation with the largest volume density of RGCs. In brief, to achieve single-localized stimulation, the current density distribution must satisfy the triggering of RGCs within the volume of stimulation. This volume is produced by the current density distribution and must be equivalent to the volume enclosed by a single RGC. Note that a single RGC and its volume could be theoretically accommodated between two electrodes of adjacent face-to-face linear carrier elements. Therefore, if current density from the electrodes reaches the RGC threshold, it can be concluded that a single or small groups of RGCs could be activated inside the volume. When a threshold peak stimulus amplitude of 0.5 nA was applied in a single, monophasic linear decrease pulse shape of 100 µs, the change density injection reached 5.66 × 10^−5^ mC/cm^2^. Sensitive retinal tissue exposure caused by electrochemical reactions is safeguarded by injecting low thresholds for efficient stimulation. Small-sized electrodes require high charge density, which can cause the breakdown of the electrode, adverse tissue reactions, and gas bubbling evolution, which can harm the layers of retinal tissue [52]. The charge injection capacity for bulk platinum is 0.1–0.35 mC/cm^2^. Platinum gray can be injected up to 1 mC/cm^2^, and titanium nitride can be injected up to 0.9 mC/cm^2^, which signifies a key step for implantable bioelectronics [23]. Brummer [52] suggested that for longer pulses, charge densities of up to 0.30–0.35 mC/cm^2^ are safe for avoiding adverse electrochemical reactions at platinum electrodes. A single, monophasic linear decrease pulse shape of 100 µs is used. The evidence from transcutaneous stimulation [53] indicates that a linear decrease pulse shape results in lower charge density, dissipated energy, and voltage across electrodes than a rectangular pulse shape. According to [15] and [54], pulses less than 150 µs in duration can induce single action potentials, light-evoked patterns, and a considerable preference to prevent the initiation of passing RGC axons. It is believed that the detailed stimulation of very-near and deep RGCs, as opposed to 2D carriers, can be achieved with the innovative technology of 3D electrode carriers. The current distribution resulting from a low threshold current generates a small volume comparable to the volume confined by individual cells. Therefore, the accurate stimulation of any given cell in the vertical segment of the ganglion layer can be realized by a low threshold stimulus, avoiding the production of electrochemical reactions. This can be helpful for the truthful restoration of natural RGC activity [14,15,16,24,25,55].

### 4.9. Existing Perforating Electrodes 

With respect to the technology of sophisticated minimally invasive visual electrode carriers (miRIs), Gerding and colleagues clearly demonstrated in several publications that perforating electrodes can certainly be implanted with no impediments and are well tolerated in prolonged-term observation [56,57,58,59,60]. Preliminary data in [56] clearly demonstrated the viability of an extraocular retina prosthetic device with posterior segment stimulation electrodes penetrating the sclera, choroid, and retina. Small-diameter electrode implants lower than 200 µm did not cause regional proliferation in the eyes of seven of the nine rabbits during a postoperative period of up to 10 months. As the preliminary results of ab externo electrode implantation were satisfactory, a definitive miRI was developed that enabled the penetration of electrodes through the sclera, chorioidea, and retina. Very slow electrode penetration was applied as a solution to prevent traumatic ad hoc penetration force. As projected, considerations such as the number and density of electrodes were demonstrated to be crucial for strictly restricting the range of related array designs. The microelectrodes penetrated the posterior segment in four of the five animals during the 6-month observation period. No retinal detachment was detected [57]. In vitro and in vivo implantation as a standard surgical procedure was developed to test the biocompatibility of implantation and long-term tissue reactions toward implants in nonhuman monkeys [58]. miRI experimental results with penetrating stimulation electrodes clearly demonstrate that this method is a promising technological alternative and was revealed to be very non-traumatic [57,59].

### 4.10. Comparison with Literature 

Several studies related to the prediction of phosphenes while the retina is electrically stimulated have been published before. Beyeler [61] demonstrated that the topographic organization of optic nerve fiber bundles in each subject’s retina can accurately predict elicited percepts using ophthalmic fundus imaging and computational modeling, allowing us to successfully replicate visual percepts that vary in orientation, from “blobs” to “streaks” and “wedges”, depending on the retinal location of the stimulating electrode. According to Greenwald [62], visual brightness may be expressed as a power function of the degree of stimulation. Additionally, brightness matching between electrodes may be predicted using the same model. Based on the neurophysiological architecture of V1, Fine [17] described a computational model, or “virtual patient”, that accurately predicts participants’ perceptual experiences across a broad range of previously published human cortical stimulation studies describing the location, size, brightness, and spatiotemporal shape of electrically induced human percepts. Since the size and shape of electrically stimulated phosphenes depend on several factors, including the size of the electrode and the current spread, our simulation findings are in agreement with those found before. Because individual neurons’ capacitance and resistance differ depending on their size, shape, and myelination, so does the sensitivity of their activating function. As the current amplitude of a pulse grows, an increasing number of cells under the electrode exceed their depolarization threshold. Increasing current amplitude causes cells further away from the electrode to exceed their depolarization threshold due to current spread, hence expanding the size of the phosphene [17].

## 5. Conclusions

Our simulation-based findings were used to identify differences between the visual sensations induced by a 2D hexagon-shaped array reported in clinical studies [5] and a patented 3D electrode array. The 2D array showed drawbacks during stimulation, like the state-of-the-art 2D visual prostheses, which provide only dot-like visual sensations in close proximity to the electrode. The 3D design could offer a technique for improving cell selectivity in epi-retinal stimulation. The reason is that low-intensity threshold activation results in volumes of stimulation equal to the volume surrounded by a solitary RGC. Our simulation framework indicates that the stimulation of an RGC can deliver stimuli below the limits of electrochemical reactions. Moreover, the algorithm shown here which estimates the shape and breadth of phosphenes can be classified as a useful tool for understanding the response of RGCs to a set of stimulating parameters, estimating the size and shape of visual percepts via an arbitrary electrode array and providing insight into the underlying brain processes, allowing for the development of analytical models of evoked percepts.

## Figures and Tables

**Table 1:** Description of the values in simulations (2D hexagonal array).

Layer	Conductivity [S/m]/Relative Permittivity [-]	Value
Polyimide Carrier	1 × 10^−17^/1	65 μm (depth)
Vitreous Humor	1.5/98	22 mm (diameter)
Epithelium Layer	2 × 10^−3^/1	65 μm (depth)
Photoreceptor Layer	28.5 × 10^−3^/1	200 μm (depth)
Intracellular Space	0.1/3.98 × 10^−11^	30 μm (depth)
PEDOT-NaPSS Coating	400/1	0.2 μm (depth)
Contact Conductivity	321/-	-
Cell Membrane	1 × 10^−8^/8.8 × 10^−11^	0.001 μm (depth)
Ganglionic Layer	0.1/1	65 μm (depth)

**Table 2:** Geometric values of the retina.

Layer	Inner Surface Diameter	Outer Surface Diameter
Epithelium	22.265 mm	22.33 mm
Photoreceptor	22.065 mm	22.265 mm
Ganglionic	22 mm	22.065 mm
Vitreous Humor	-	22 mm

**Table 3:** Description of the parameters in simulations (3D electrode carrier).

Parameters	Conductivity [S/m]/Relative Permittivity [-]	Values
Polyimide Carrier	1 × 10^−17^/1	15 μm (depth)
LCE ^3^ Length		60 μm
LCE Depth		2 μm
LCE Outer Diameter ^1^		9 μm
LCE Width ^2^		5 μm
Inner Nuclear Layer	15 × 10^−3^/1	100 μm (depth)

^1^ Outer electrode diameter. ^2^ Width between electrodes. ^3^ LCE is linear carrier element.

## Data Availability

The algorithm presented in this study is available on request from the corresponding author.
